# Out of Balance: R-loops in Human Disease

**DOI:** 10.1371/journal.pgen.1004630

**Published:** 2014-09-18

**Authors:** Matthias Groh, Natalia Gromak

**Affiliations:** Sir William Dunn School of Pathology, University of Oxford, Oxford, United Kingdom; University of Washington School of Medicine, United States of America

## Abstract

R-loops are cellular structures composed of an RNA/DNA hybrid, which is formed when the RNA hybridises to a complementary DNA strand and a displaced single-stranded DNA. R-loops have been detected in various organisms from bacteria to mammals and play crucial roles in regulating gene expression, DNA and histone modifications, immunoglobulin class switch recombination, DNA replication, and genome stability. Recent evidence suggests that R-loops are also involved in molecular mechanisms of neurological diseases and cancer. In addition, mutations in factors implicated in R-loop biology, such as RNase H and SETX (senataxin), lead to devastating human neurodegenerative disorders, highlighting the importance of correctly regulating the level of R-loops in human cells. In this review we summarise current advances in this field, with a particular focus on diseases associated with dysregulation of R-loop structures. We also discuss potential therapeutic approaches for such diseases and highlight future research directions.

## Introduction

**Figure 1:**
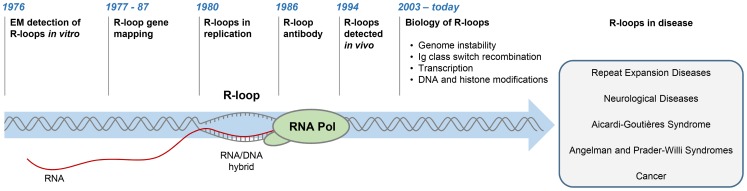
History of R-loop research. The diagram depicts major developments in the R-loop field and diseases associated with R-loop dysregulation.

R-loops are three-stranded structures, which form when RNA hybridises to a complementary DNA strand, forming an RNA/DNA hybrid, resulting in displacement of the other DNA strand in this process (Figure 1). The first R-loops were described in 1976, when their formation in vitro in the presence of 70% formamide was visualised by electron microscopy (Figure 1) [1]. These structures were thermodynamically more stable than duplex DNA, and they remained intact following removal of formamide. This technique of RNA/DNA hybridisation has been used in over 140 studies to map gene organisation, transcription initiation sites, and the direction of transcription, as well as measure the quantities of cellular RNAs [2].

The first evidence for R-loop formation in live bacteria was obtained in 1994 [3]. This was followed by numerous studies showing that R-loops exist in different organisms (Figure 1) [4]–[6]. In living cells, R-loops are thought to form in cis during transcription, when nascent RNA hybridises to the DNA template behind the elongating RNA polymerase (Pol) [4]. However, in contrast to this popular view of cotranscriptional R-loops, recent studies suggest that RNA transcribed at one locus can hybridise to homologous DNA at another locus, thus leading to R-loop formation in trans [7]. In the last five years, the use of an antibody (S9.6) recognising RNA/DNA hybrids has revolutionised the R-loop field [8]. Initially, the S9.6 antibody, which detects hybrids as small as six bp with an affinity of 0.6 nM, was developed as a tool to enhance the DNA/RNA hybridisation signal in DNA microarray studies [9], [10]. More recently, it has been used to detect R-loops in vivo and uncover their contribution to fundamental biological processes in yeast [11], [12], plants [13], mice [14], [15], and humans [16]–[18].

The picture emerging from these studies suggests that R-loops can be both beneficial and deleterious to cells. Paradoxically, while they are required for important biological processes, they can also promote DNA damage and genome instability. In particular, R-loops have been shown to play an essential positive function in *Escherichia coli* plasmid and human mitochondrial DNA replication [19], [20] and during immunoglobulin class switch recombination, which contributes to the antibody isotype diversity in activated B cells [21]. R-loops form on many genes in yeast and human cells [18], [22] and have been implicated in regulation of gene expression. R-loops can repress transcription and promote transcriptional termination [16], [23], [24]. Furthermore, R-loops are clearly associated with epigenetic mechanisms governing transcription, including DNA methylation and posttranslational histone modifications [18], . In spite of this growing list of beneficial R-loop functions, it is also evident that R-loops can be a dangerous source of DNA damage. They can sensitize DNA to damaging agents [28], induce transcription-associated recombination [24], double-strand breaks (DSBs) [29], [30], chromosome breaks, and fragile site instability [31]–[33], and cause chromosome loss [34]. Therefore, cells need to tightly regulate the levels of R-loops to exploit their unique features. Altering the physiological R-loop balance can impair R-loop-regulated processes, cause genome instability, and may lead to human diseases. Consequently, defining the roles of R-loops in the multitude of biological processes and human disease is likely to develop into one of the most important and influential areas of R-loop research in the future.

## Proteins in R-loop Biology

The number of proteins associated with R-loop biology has increased in the last few years, reflecting the diversity of R-loop processes (Table S1) [4]–[6]. Many proteins can regulate cellular R-loop levels either directly or indirectly, mostly by preventing RNA from hybridising to DNA, thus reducing excessive R-loop accumulation. Among these are proteins required for efficient transcriptional elongation, termination, polyadenylation, RNA splicing, packaging, and export [16], [24], [28], [30], [31], [34], [35]. DNA topology itself can influence hybridisation of RNA to DNA, and topoisomerases consequently play important roles in modulating R-loop levels [27], [33]. Proteins involved in maintenance of genome integrity can also regulate R-loops, suggesting a dynamic interplay between DNA repair and R-loop formation [7]. Importantly, cells possess dedicated enzymes, including the members of the RNase H family that specifically degrade the RNA in R-loops [36], and helicases that can unwind RNA/DNA hybrids [12], [16].

Recent evidence shows that R-loops can directly affect many gene expression–associated processes, including DNA methylation, posttranslational histone modifications, and transcription, by influencing the function of regulatory proteins [16],[18],[25],[26]. Despite the growing number of proteins involved in R-loop homeostasis and human disease, many questions still remain unanswered. For many proteins with documented in vitro RNA/DNA helicase activity (e.g., Pif1, the MCM complex), in vivo evidence is generally still lacking (Table S1) [37], [38]. Moreover, the molecular mechanisms underlying interactions between proteins and R-loops are poorly understood, and in many cases the connections to disease remain obscure.

## R-loops and Neurological Diseases

**Figure 2:**
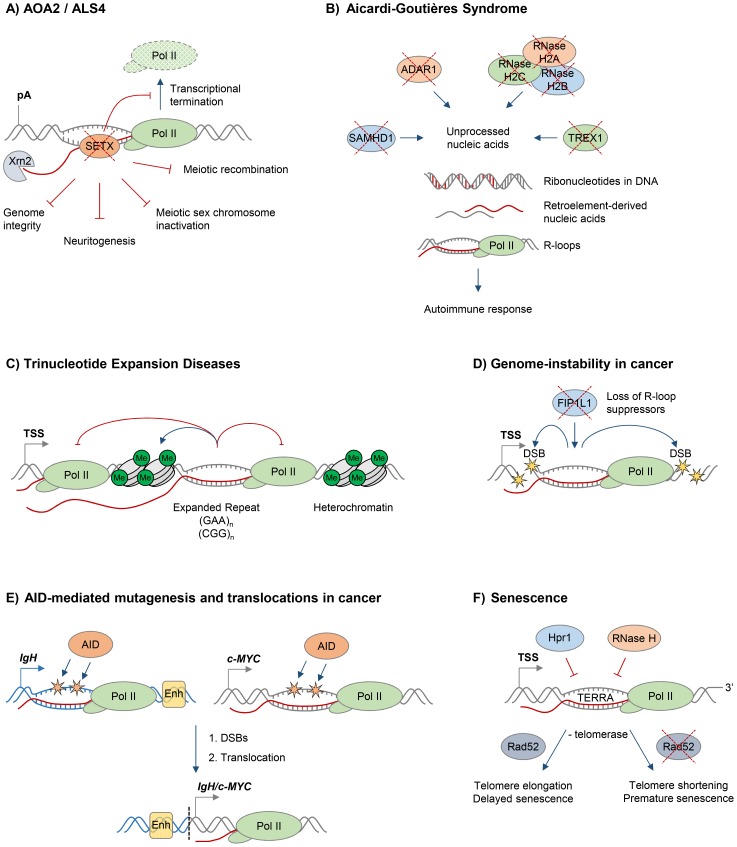
R-loops and human diseases. The diagram depicts the role of R-loops in human diseases. Loss of wild type protein function is depicted by red crosses. **A.** Ataxia and motor neuron diseases. Mutations in human RNA/DNA helicase senataxin are associated with AOA2/ALS4 disorders and lead to R-loop accumulation and defects in transcriptional termination by Pol II [16], the maintenance of genome integrity [46], meiotic recombination during spermatogenesis, gene silencing during meiotic sex chromosome inactivation [14], and neuronal differentiation [49]. **B.** Aicardi-Goutières syndrome (AGS). AGS is associated with mutations in all three subunits of RNase H2, ssDNA 3′–5′ exonuclease TREX1 (DNASEIII), dsRNA-editing enzyme ADAR1, and dNTP triphosphatase SAMHD1; these trigger accumulation of unprocessed nucleic acids, including genomic DNA with incorporated ribonucleotides, R-loops, and retroelement-derived nucleic acids, and result in the immune response characteristic of AGS [65]. **C.** Trinucleotide expansion diseases. R-loops form over expanded repeats and result in decreased initiation and elongation of RNA Pol II and formation of repressive chromatin marks, which silence the host gene containing expanded repeats [75]. **D.** Genome instability in cancer. Loss of proteins protecting against abnormal R-loop accumulation, such as FIP1L1, leads to genome instability, one hallmark of cancer [31]. Yellow stars denote double-stranded DNA breaks. **E.** AID-mediated mutagenesis and translocations in cancer. Single-stranded DNA in R-loops is a substrate for cytidine deamination by activation-induced cytidine deaminase, leading to mutagenesis as indicated by orange stars [21], [88]. These mutations can cause DSB formation, leading to chromosomal translocations. The *IgH/c-MYC* translocation brings the strong *IgH* enhancers, shown as yellow box, close to *c-MYC*, leading to its overexpression in Burkitt's lymphoma [87]. Transcription of *IgH/c-MYC* starts from a previously inactive promoter downstream of the translocation break point. The *IgH* locus is depicted in blue, *c-MYC* gene is in grey. The translocation breakpoint is indicated by a dashed black line. **F.** Senescence. R-loops formed by the noncoding RNA TERRA accumulate at telomeres in cells deficient of Hpr1 and RNase H. In the absence of telomerase, these R-loops promote Rad52-dependent telomere elongation and delayed senescence. In the absence of telomerase and Rad52, R-loops promote telomere shortening and premature senescence [94].

The biological importance of R-loops in humans is supported by the fact that mutations in proteins implicated in R-loop resolution cause devastating human diseases, often related to neurodegeneration. Mutations in the putative RNA/DNA helicase SETX cause neurodegenerative diseases, the dominant juvenile form of amyotrophic lateral sclerosis type 4 (ALS4), and a recessive form of ataxia oculomotor apraxia type 2 (AOA2) (Figure 2A). These diseases are characterised by progressive degeneration of motor neurons in the brain and spinal cord, muscle weakness and atrophy [39]–[41].

In addition to its predicted function as an RNA/DNA helicase, SETX interacts with proteins involved in diverse aspects of RNA metabolism [42]. Moreover, a single amino acid mutation, which compromises the function of the yeast homologue Sen1, dramatically changes the Pol II distribution genome-wide, further supporting the view that SETX/Sen1 functions in the regulation of transcription [43]. Recently, we demonstrated that SETX is implicated in transcriptional termination by Pol II in humans [16]. It is required to resolve R-loops at termination elements, releasing RNA for degradation by the 5′–3′ “torpedo” exonuclease Xrn2 prior to termination (Figure 2A) [16], [44]. Mutations in the yeast homologue, Sen1, also lead to a transcriptional termination defect, associated with accumulation of R-loops and genome instability [12]. In line with its function in R-loop resolution, SETX/Sen1 is also involved in maintaining genome integrity by coordinating transcription, DNA replication, and the DNA damage response [45]–[47]. SETX can target the 3′–5′ RNA degradation complex, the exosome, to sites of transcription-induced DNA damage [48]. Furthermore, SETX protects genome integrity by coordinating meiotic recombination with transcription during spermatogenesis and gene silencing during meiotic sex chromosome inactivation [14]. In particular, *Setx* knock-out mice accumulated DNA double strand breaks and R-loops and failed to disassemble Rad51 filaments. This resulted in a failure to cross over, likely due to collision between R-loops with Holliday junctions [14]. These defects in *Setx* knock-out mice lead to male infertility, raising the question as to how this relates to fertility of male AOA2/ALS4 patients.

Studies in neuronal cells have demonstrated a role for SETX in neuronal differentiation through fibroblast growth factor 8 (FGF8) signalling, providing one explanation for the effects of loss-of-function AOA2 mutations [49]. Surprisingly, overexpression of dominant mutant forms of SETX did not affect neuritogenesis, suggesting that a different function of SETX may be affected in ALS4 patients. However, the interplay between the function of SETX in R-loop resolution, genome maintenance, and neuronal differentiation is still unclear. In a recent study, Lavin and colleagues examined cells from mice with disrupted *Atm*, *Tdp1*, *Setx*, or *Aptx* genes, which cause ataxia telangiectasia (AT), spinocerebellar ataxia with axonal neuropathy 1 (SCAN1), AOA2, and ataxia oculomotor apraxia type 1 (AOA1) disorders, respectively [15]. These diseases are characterised by a defective response to DNA damage, suggesting that R-loops may be implicated in triggering genome instability. Indeed, R-loops were found to be enriched in proliferating cells (testes), but not in the brain tissues from *Setx*, *Atm*, *Tdp1* or *Aptx* knock-out mice [15]. The enrichment of R-loops in testes correlated with high levels of DNA damage and apoptosis. The lack of R-loops in brain tissue questions the association between R-loops and neurodegeneration. This result is surprising, because inducible R-loops have been previously detected in neuronal cells at the *Snord116* locus, which is associated with the neurodevelopmental disorder Angelman syndrome, as discussed below [50]. Furthermore, R-loops were implicated in inducing DNA damage in nonproliferating cells and post-mitotic neurons and proposed to contribute to the neurodegeneration seen in AT patients [29]. It is possible that R-loops are regulated by different mechanisms in proliferating cells and post-mitotic neurons, thereby leading to different R-loop kinetics and so preventing their detection in some model systems. In particular, R-loop accumulation may reflect collisions between transcription and replication machineries [32], [51], events which do not occur in postmitotic neurons. It should be noted that the mouse models currently used may not fully recapitulate all aspects of human neurodegeneration.

## RNase H and Aicardi-Goutières Syndrome (AGS)

In addition to their generation during transcription, RNA/DNA hybrids can arise due to incorporation of ribonucleotides into DNA by DNA polymerases during replication. RNase H enzymes are endonucleases that cleave the RNA of RNA/DNA hybrids in a sequence-independent manner, thus maintaining genome stability by resolving R-loops that form during transcription and by removing misincorporated ribonucleotides from the DNA [36]. Eukaryotic cells have two types of these enzymes, RNase H1 and RNase H2, which have different enzymatic and site-specific activities [52]. In particular, RNase H1 requires a tract of at least four ribonucleotides to cleave the RNA/DNA hybrid, whereas RNase H2 can incise 5′ to a single ribonucleotide incorporated within a DNA molecule [36], [52]. Therefore, only RNase H2 can process single ribonucleotides in the DNA, but both enzymes are capable of eliminating RNA/DNA hybrids. Unlike in bacteria and unicellular eukaryotic organisms, where RNase H enzymes are dispensable for viability, both RNase H enzymes are essential in higher eukaryotes. RNase H1 has been implicated in mitochondrial DNA (mtDNA) replication during mouse development, a process likely to be associated with processing of RNA primers during mtDNA replication [53].

RNase H2 is composed of three different subunits, the catalytic subunit 2A, and two other subunits, 2B and 2C, all of which are required for enzyme activity. RNase H2 has been implicated in recognition and removal of ribonucleotides incorporated into DNA and hydrolysis of Okazaki fragment RNA primers during DNA replication [36], [54]–[57]. In addition, recent studies point towards a role of RNase H2 in R-loop resolution during transcription in vivo [11], [58]. In particular, deletion of *Saccharomyces cerevisiae* RNase H2 imposes transcriptional blocks and R-loop accumulation over rDNA regions in cells depleted of Topoisomerase I [11] and transcriptional down-regulation of genes with higher guanine-cytosine (GC) content at the promoter regions, which are likely to form stable R-loops [58].

In humans, mutations in any of the three subunits of RNase H2 cause Aicardi-Goutières syndrome (AGS), a neurological inflammatory disorder, which resembles a congenital viral infection and is associated with accumulation of ribonucleotides in the DNA (Figure 2B) [59], [60]. Interestingly, AGS can also be triggered by mutations in single-stranded DNA (ssDNA) 3′–5′ exonuclease TREX1(DNASEIII) [61], double-stranded RNA (dsRNA)-editing enzyme ADAR1 [62], and dNTP triphosphatase SAMHD1[63]. These proteins are involved in diverse pathways of nucleic acid metabolism, although their functions are not yet fully understood. They have been implicated in degrading ssDNA arising from endogenous retroelements or replication stress (TREX1), regulating the intracellular dNTPs pool available for replication and reverse transcription of these retroelements (SAMHD1), or altering the immune response to RNA species through RNA editing of retroelements and microRNAs (ADAR1) [64]. Mutations in these proteins are associated with an accumulation of unprocessed nucleic acids, which triggers the immune response characteristic of AGS [64], [65].

So far, pathologies linked to AGS mutations in RNase H2 have been mainly attributed to genome instability caused by accumulation of ribonucleotides in DNA [56], [66]. However, a specific contribution of R-loops and RNA/DNA hybrids to AGS pathology has not been yet investigated. This research has been hampered by the difficulty to uncouple the two activities of RNase H2; its ability to remove ribonucleotides from the DNA and to resolve R-loops, both of which are affected when RNase H2 is deleted [52], [56]. Nevertheless, several lines of evidence suggest that R-loops may be involved in AGS pathology. Thus, an AGS-related mutation in the yeast RNase H2 enzyme resulted in its reduced RNA/DNA cleavage activity [52]. Since RNase H2 constitutes ∼90% of the total cellular RNA/DNA hybrid cleavage activity, its loss due to AGS mutations may lead to significant accumulation of R-loops [56]. The importance of RNase H2 is further highlighted by the fact that mutations in RNase H1 do not cause AGS, suggesting that RNase H2 may have unique properties to degrade RNA/DNA hybrids [52]. Indeed, R-loops arising during DNA replication may be exclusively degraded by RNase H2, as they may be inaccessible to RNase H1 [52], [67]. A recently generated *S. cerevisiae* RNase H2 mutant, which possesses R-loop degrading activity but fails to remove single ribonucleotides from the DNA [52], will be a useful tool in addressing the contribution of unresolved transcription-associated R-loops to AGS pathology.

TREX1, ADAR1 and SAMHD1 process retroelement-derived nucleic acids and help to suppress retroelements expansion in the host genome and their recognition by the immune system [64]. Interestingly, recent genome-wide studies have demonstrated that RNA/DNA hybrids are particularly enriched at retrotransposon elements in yeast cells [22], suggesting that expansion of retroelements due to mutations in TREX1, ADAR1 or SAMHD1 may lead to increased RNA/DNA hybrid levels, contributing to autoimmunitity in AGS. Indeed, it has recently been demonstrated that RNA/DNA hybrids can be sensed by toll-like receptor 9 (TLR9) to induce pro-inflammatory cytokine and antiviral interferon production in dendritic cells [68].

## R-loops in Nucleotide Expansion Diseases

Expansions of repetitive sequences have been linked to over forty human diseases [69], and R-loops have been proposed to play a role in their pathology [70]–[73]. Remarkably, R-loops are formed following transcription of trinucleotide repeats in vitro, in bacteria and human cells [70], [71], [73]. Interestingly, the nontemplate DNA strand in many repetitive sequences can adopt unusual DNA structures, including G-quadruplexes and DNA triplexes, which may further stabilise R-loops [74]. Moreover, R-loops formed at CTG repeats promote repeat instability characteristic of these diseases [71].

Recently, we demonstrated that R-loops form over expanded GAA and CGG repeats in cells from Friedreich's Ataxia (FRDA) and Fragile X syndrome (FXS) patients, respectively (Figure 2C) [75]. The abundance of these stable R-loops correlates with expansion size, and they colocalise with the repressive chromatin marks characteristic of these diseases (Figure 2C). R-loops can also trigger the formation of repressive chromatin and cause transcriptional silencing of the *FXN* gene, providing a molecular link between R-loops and the pathology of expansion diseases [75]. In line with R-loops formed on expanded “premutation” and “full mutation” CGG-repeat-containing alleles of the *FMR1* gene [75], [76], promoter-bound *FMR1* mRNA containing trinucleotide repeats was shown to promote epigenetic silencing in FXS [77]. Importantly, the involvement of R-loops in expansion diseases is not limited to trinucleotide repeats, since R-loops associated with expanded hexanucleotide GGGGCC repeats in C9orf72 contribute to the molecular event leading to amyotrophic lateral sclerosis (ALS) and frontotemporal dementia (FTD) [78].

R-loops could contribute to the pathology of expansion diseases in various ways. Similar to R-loops at the 3′ends of human genes, expansion-associated R-loops may form a structural block, directly interfering with Pol II transcriptional elongation [16], [24]. Alternatively, R-loops may nucleate repressive chromatin over the expansion region, by analogy with heterochromatin formation at centromeres in *Schizosaccharomyces pombe* [25], or promote chromatin compaction associated with histone H3S10 phosphorylation, as observed in *S. cerevisiae*, *Caenorhabditis elegans*, and human cells [26]. Furthermore, R-loops could cause the characteristic intergenerational and somatic instability of repeat sequences [72].

## R-loops in Cancer

Genome instability is a hallmark of cancer, and it may actively drive hereditary tumour development [79], [80]. Research in the last decade has clearly demonstrated that dysregulation of R-loops can corrupt genome integrity, resulting in increased DNA sensitivity to damaging agents, formation of DSBs, chromosome breaks, fragile site instability, chromosome loss, and recombination events [5]. Several mechanisms have therefore evolved to maintain R-loop levels in balance, and alterations in genome caretaker processes can affect R-loop levels and genome stability [4]. Moreover, mutations in proteins controlling R-loop levels have been identified in tumours (Figure 2D). For example, in eosinophilic leukemia, an oncogenic translocation renders cleavage and polyadenylation factor FIP1L1 inactive, which has been previously shown to cause increased R-loop levels, DNA damage and chromosome instability (Figure 2D) [31]. A similar mechanism was suggested for RNA kinase CLP1, which is associated with a translocation in mixed lineage leukemia (MLL) [31]. The histone ubiquitin ligase BRE1 also limits R-loop levels, and its decreased expression may contribute to the high levels of genomic instability observed in testicular seminoma [81].

The link between R-loops and cancer has been further substantiated by the finding that the tumour suppressor BRCA2, which is mutated in breast and ovarian cancer, is required to prevent R-loop accumulation and genome instability [82]. These observations raise the interesting possibility that R-loops may provide proliferative advantages to tumour cells by promoting genome instability. This will in turn increase the probability of accumulating mutations favourable to tumour growth and metastasis. Intriguingly, recent evidence demonstrates that human oncogenic viruses may also promote genomic instability through accumulation of R-loops after infection. Kaposi's sarcoma-associated herpesvirus (KSHV), which causes multiple AIDS-related cancers, encodes the ORF57 protein, which can sequester the host hTREX complex, important for mRNA processing and export [83]. Sequestration of hTREX leads to KSHV-induced accumulation of R-loops and causes damage to the host DNA, contributing to tumourigenesis [83].

Whilst some proteins suppress R-loop formation, others may promote R-loops and so increase genome instability leading to tumour development. This unexpected function has been shown in yeast for transcription elongation factor Spt2 and DNA repair protein Rad51 [7], [84]. Overexpression of Spt2 leads to transcription-dependent chromosomal rearrangements, which are prevented by RNase H overexpression [84]. Spt2 is structurally related to human HMG1, which is overexpressed in gastric cancers and malignant melanomas [84]. However, it is not clear if increased HMG1 levels promote R-loops and DNA damage in cancer cells. In contrast to its well-established role in DNA strand exchange during homologous recombination and DNA repair [85], recent studies have shown that Rad51 can also mediate R-loop formation and genome instability in trans, extending the prevailing view that R-loops form cotranscriptionally [7]. Similar to HMG1, RAD51 is overexpressed in human cancers [7]. However, it remains to be elucidated if RAD51 overexpression in cancers is a consequence of activated DNA repair pathways, or a cause of genome instability [7].

R-loops have been detected in immunoglobulin (Ig) genes, where they initiate class switch recombination by exposing single-stranded DNA, thus providing the substrate for activation-induced cytidine deaminase (AID), which promotes DSBs and subsequent translocation between Ig heavy chains [21], [86]. Although this process is essential for generation of antibody isotype diversity, AID-mediated mutagenesis has also been implicated in pathological translocations between the Ig loci and other active genes, leading to production of fusion proteins or oncogenic gene expression, observed in B cell malignancies (Figure 2E) [87]. Interestingly, R-loops are also found in common translocation partners of Ig genes, including the oncogene *c-MYC* [18], [27]. Therefore, the simultaneous formation of R-loops in Ig and transcribed non-Ig genes may induce AID-mediated DSB formation, leading to pathological translocations (Figure 2E) [27], [88], [89]. Interestingly, overexpression of the APOBEC family of AID-related enzymes in breast cancer have been linked to genomic mutations, pointing to a potentially broader role of R-loops and AID/APOBEC-mediated genome instability in cancer [90].

Changes in gene expression are another central aspect of cancer [79]. In healthy cells, the expression of tumour suppressor genes prevents abnormal proliferation and other aspects of tumourigenesis [79]. Tumour suppressors are frequently silenced in cancer by excessive promoter DNA methylation [91]. It has been proposed that R-loop formation at promoters protects against DNA methylation by de novo DNA methyltransferase DNMT3B, thereby keeping genes active [18]. Since R-loops have been computationally predicted to form at promoters of tumour suppressor genes *BRCA1*, *RASSF1A*, and *CDKN2A* [92], it is important to investigate if R-loop levels at these genes are reduced in cancer and how this relates to the observed DNA hypermethylation.

In contrast to this, efficient transcription of the oncogene *c-MYC* requires that R-loop levels are kept low by the activity of DNA topoisomerase IIIB, which is recruited to arginine-methylated histones by the tudor domain containing 3 (TDRD3) protein [27]. This R-loop-mediated mechanism of *c-MYC* gene regulation may be relevant to tumour progression in breast cancer, which frequently shows overexpression of both *c-MYC* and TDRD3 [27], [93]. Therefore, it is tempting to speculate that increased TDRD3 levels suppress R-loops in *c-MYC*, thereby allowing its enhanced expression, which correlates with poor cancer prognosis [93]. However, it still remains to be determined if R-loops play a specific role in transcription dysregulation in cancer and if this process differs from R-loop-mediated transcriptional programmes associated with housekeeping genes.

More recently, R-loops have been implicated in cell senescence, a mechanism protecting against tumour cell proliferation [79]. In particular, the telomeric noncoding (nc) RNA TERRA forms R-loops which are induced when R-loop suppressors such as RNase H or Thp2 are lost [94], [95]. In the absence of telomerase, telomeric R-loops promote recombination-mediated telomere elongation via Rad52, and this delays the onset of cellular senescence [94]. In contrast, in Rad52-deficient cells, R-loop accumulation leads to telomere shortening and premature senescence [94]. Interestingly, cells from AOA2 patients with senataxin mutations contain shorter telomeres, suggesting a possible involvement of SETX in telomere stability [96]. Telomeric R-loops therefore play a complex and dynamic role in telomere length maintenance and cellular proliferative potential (Figure 2F).

In conclusion, multiple lines of evidence point to an involvement of R-loops in cancer biology. Yet it still remains to be investigated if R-loop levels are indeed regulated differentially in normal and tumour tissues and if they can directly influence tumourigenesis.

## R-loop Therapies

R-loops represent a potential therapeutic target. Despite their importance in gene regulation, they have yet to be fully exploited in drug design [97]. Various ligands can target RNA/DNA hybrids, including ethidium bromide, the aminoglycosides neomycin and paramomycin, and the polyamides distamycin and netropsin [98]. These compounds recognise RNA/DNA hybrids through intercalation and binding to the nucleic acid groove. Although exhibiting high binding affinities to RNA/DNA hybrids, many of these molecules also bind dsDNA and RNA and are mutagenic, limiting their potential biological applications [98]. However, recent studies suggest that combining the properties of these ligands can achieve subnanomolar affinity for RNA/DNA hybrids. In particular, this has been demonstrated for ligands linking aminoglycosides to derivatives of ethidium bromide [99], providing a possible approach for the development of potent and specific RNA/DNA hybrid ligands in future drug design efforts.

**Figure 3:**
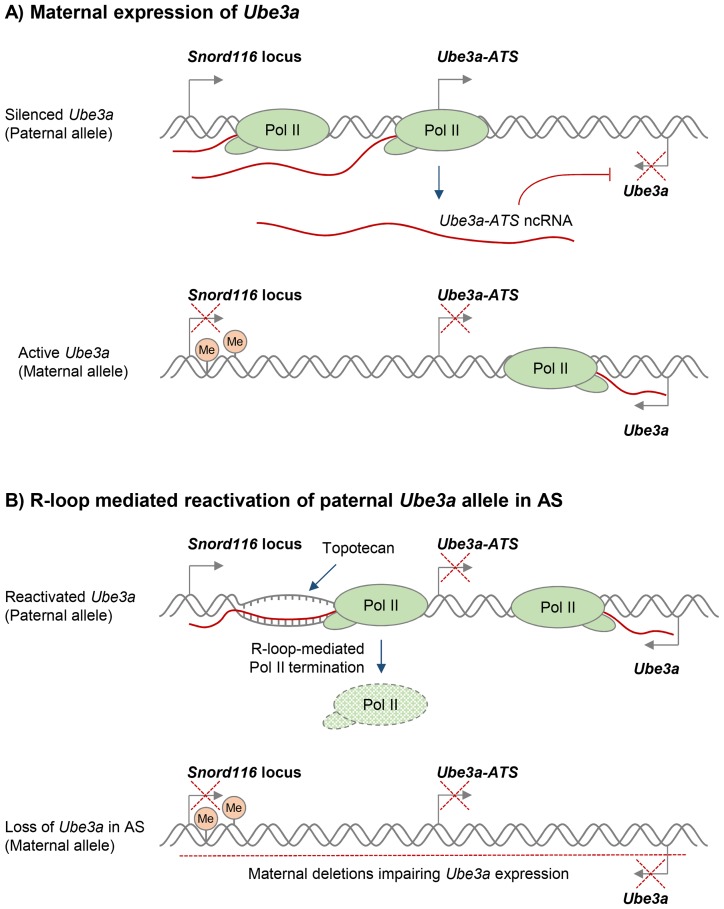
Potential R-loop-based therapeutic approach in Angelman Syndrome (AS). **A.** Neuronal expression of the paternal ncRNA *Ube3a-ATS* represses paternal *Ube3a* gene in cis [101]. DNA methylation of the *Snord116* locus on the maternal allele prevents *Ube3a-ATS* transcription, resulting in *Ube3a* expression from the maternal allele. Transcriptional repression is indicated by red crosses. **B.** R-loop-mediated re-activation of silent paternal *Ube3a gene* provides a targeted therapy for AS. Deletion leading to the loss of maternal *Ube3a* expression detected in AS is indicated by the red dashed line. Topotecan treatment increases R-loop levels over the *Snord116* locus, resulting in chromatin decondensation, inhibition of Pol II transcription through *Ube3a-ATS*, and increased expression of *Ube3a* from the paternal allele [50].

Various compounds that modulate DNA supercoiling and inhibit DNA topoisomerases, including topotecan and camptothecin, can also affect R-loop formation in vivo [29], [50]. In particular, topoisomerase inhibitors have recently been used to reactivate the silenced paternal *Ube3a* gene, which encodes a ubiquitin E3 ligase, to compensate for the deleted maternal *Ube3a* in Angelman syndrome (AS). AS and Prader-Willi syndrome (PWS) are imprinted neurodevelopmental disorders that are often caused by large deletions of human chromosome 15q11–q13 over the *Snord116* gene locus, but the deletion differs in its parent-of-origin [100]. In neurons, only the maternal *Ube3a* allele is expressed, because the paternal *Ube3a* allele is silenced by expression of the ncRNA *Ube3a-ATS* (Figure 3A) [101]. AS therapies therefore seek to reactivate the silenced, but genetically intact, paternal *Ube3a* allele. Interestingly, R-loops were recently shown to regulate the neuronal expression of the paternal *Ube3a-ATS* transcript, which is essential for transcriptional silencing of the paternal *Ube3a* gene [50]. In particular, treatment with the topoisomerase inhibitor topotecan increased R-loop levels over the *Snord116* locus, resulting in chromatin decondensation, inhibition of Pol II transcription of *Ube3a-ATS*, and concomitant increase in *Ube3a* expression from the paternal allele (Figure 3B). This R-loop-mediated reactivation of paternal *Ube3a* could therefore compensate for the loss of maternal *Ube3a* in AS and so potentially holds promise for targeted therapies for both AS and PWS (Figure 3B).

It has previously been proposed that R-loops in trinucleotide expansion diseases could be targeted to suppress repeat expansions or reactivate silenced genes [72]. A recent study provided direct evidence that a small molecule is able to suppress R-loop formation at expanded CGG repeats in the *FMR1* gene, thereby preventing *FMR1* epigenetic silencing in FXS [77]. As an alternative approach, R-loop levels may be indirectly modulated by treatments that target proteins involved in R-loop biology (Table S1). For instance, genomic instability caused by a widespread increase of R-loops due to loss of an R-loop suppressing protein could potentially be reverted by introduction of an alternative R-loop suppressor.

Recent identification of small-molecule inhibitors for RNase H2 may also provide a powerful new tool for the study of R-loop biology in health and disease [102]. Furthermore, the S9.6 antibody offers new opportunities for research and development. In particular, it has already been used in the development of biosensor systems [103], detection of miRNA targets [104], and as a key component of human papillomavirus (HPV) diagnostic kit (Qiagen).

The explosion of studies uncovering the role of R-loops in health and disease in recent years provides the exciting prospect of developing new targeted therapeutics for many human disorders. However, due to the ubiquitous nature of R-loops it will be important to ensure that efficient treatments are specific.

## Conclusions and Future Challenges

R-loops have been implicated in many biological processes in different organisms. R-loops can play positive and negative roles in gene expression; they can mediate Ig class switch recombination and transcriptional termination, affect genome stability, transcription, cell cycle progression, and cell viability. Despite the diversity of these biological processes, the molecular mechanisms associated with R-loop formation in mammalian cells remain largely unknown. It is unclear how R-loops can regulate gene expression, how they are maintained and eliminated in the cells, and which proteins are involved in the regulation of these processes.

The connections between R-loops and human diseases suggest that cells have evolved mechanisms to distinguish between deleterious and beneficial R-loops. However, the evidence discussed above raises an important question: how can R-loop dysregulation be mechanistically linked to a variety of human diseases with such diverse pathologies? One explanation may be that R-loops form in many genomic locations in healthy cells [16], [18], [22], [27]. Therefore, unsurprisingly, their dysregulation can affect a large number of disease-associated genes. This is in contrast to gene-specific R-loop pathologies, associated with mutations, which result in altered R-loop levels locally, as observed in the repeat expansion diseases FRDA and FXS [75],[77]. Furthermore, R-loops can have different intrinsic properties. R-loops at expanded GAA repeats in the *FXN* gene are highly stable and trigger transcriptional repression, while R-loops in the highly-expressed *γ-Actin* gene are easily turned over [75]. This could, in part, be due to differential activity of R-loop processing proteins on different classes of genes, as proposed in yeast [22]. Adding another layer of complexity, the formation of R-loops can be influenced by cell type [77], cell cycle stage [15], gene length, and/or GC content and transcriptional level [22], [105]. Epigenetic marks including DNA methylation and post-translational histone modifications can contribute to further modulation of R-loop levels [18], [27]. Thus, R-loops represent cellular structures that share the same elementary composition, but may possess different dynamic properties, which can be affected by any of the aforementioned processes, thus explaining the wide range of diseases associated with R-loops.

Despite the lack of mechanistic insights into R-loop-associated diseases, some common themes, underlying their pathology, are already becoming obvious. First, there is a strong connection between R-loop dysregulation and induction of DNA damage and loss of genome integrity, which contributes to cancer development [31], [81], [88], repeat expansion diseases [71], and neurodegeneration [29], [45]. Secondly, R-loops can mediate changes in transcription locally or globally, contributing to pathologies associated with repeat expansion diseases [75], [77], [78], Angelman syndrome [50], and cancer [27]. However, it is a strong possibility that both of these pathological themes may overlap in many disorders, as observed in repeat expansion diseases [71], [75], [77], and novel disease themes may be revealed in the future.

One of the major challenges in R-loop field is to investigate the causes and consequences of R-loop formation in additional models of human disease. Uncovering further aspects of R-loop biology in human cells will certainly shed light on many basic biological questions and have major implications for our understanding of human disease. Future studies will undoubtedly reveal more diseases associated with R-loop dysregulation and will provide the basis for novel therapeutic approaches targeting these so far overlooked structures in gene expression.

## Supporting Information

Table S1 **Proteins implicated in R-loop biology.** For multiprotein complexes, only subunits directly implicated in R-loop biology are mentioned in the table. *Asterisk indicates that protein association with R-loops is based on in vitro evidence.
(DOCX)

Text S1 **Supplemental references.**
(DOCX)
