# Supplementary material for: Out of Balance: R-loops in Human Disease
Source: PLoS Genet. 2014 Sep 18;10(9):e1004630. doi: 10.1371/journal.pgen.1004630 (PMC4169248; doi:10.1371/journal.pgen.1004630)
Supplement: Table S1 — Proteins implicated in R-loop biology. For multiprotein complexes, only subunits directly implicated in R-loop biology are mentioned in the table. *Asterisk indicates that protein association with R-loops is based on in vitro evidence. (DOCX) [file pgen.1004630.s001.docx]

**Table S1. Proteins implicated in R-loop biology.**

| **Protein** | **Organism** | **Reference** |
| --- | --- | --- |
|  |  |  |
| **Transcription initiation and capping** |  |  |
| Bur2 | Yeast | [34] |
| Mediator kinase module (Med12, Med13, Cdk8, CycC) | Yeast | [34] |
| Mediator complex (Med1, Med5) | Yeast | [34] |
| Taf5 | Yeast | [22] |
| Mot1 | Yeast | [22] |
| Not5 | Yeast | [34] |
| Capping enzyme | Human | [106]* |
|  |  |  |
| **Transcription elongation** |  |  |
| PAF1 complex (Leo1, Cdc73) | Yeast | [7,34] |
| Spt2 | Yeast | [34,84] |
| FACT complex (Spt16/SPT16, Pob3/SSRP1) | Yeast, human | [35] |
|  |  |  |
| **Transcription termination, cleavage and polyadenylation** |  |  |
| CFIA complex (Pcf11, Clp1, Rna15) | Yeast | [31] |
| Rtt103/KUB5-HERA | Yeast, human | [31,107] |
| Sen1/SETX | Yeast, mouse, human | [12,14-16,22,26,31,46,47] |
| CPF complex (Cft2, Fip1/FIP1L1) | Yeast, human | [31] |
|  |  |  |
| **RNA processing and export** |  |  |
| THO/TREX complex (Tho2/THOC2, Hpr1/THOC1, Mft1, Thp2, Sub2/UAP56) | Yeast, nematode, mouse, human | [22,24,26,31,94,95,108-110] |
| THSC complex (Thp1/PCID2, Sac3/GANP, Sus1, DSS1) | Yeast, Human | [82,108] |
|  |  |  |
| Npl3 | Yeast | [28,34] |
| Dbp6 | Yeast | [22] |
| Dbp7 | Yeast | [22] |
| Rpf1 | Yeast | [22] |
| Imp4 | Yeast | [22] |
| Xrn1/Kem1 | Yeast | [7,34] |
| Srm1 | Yeast | [31] |
| Nup133 | Yeast | [22] |
| Rna1 | Yeast | [22] |
| Kae1 | Yeast | [22] |
| TRAMP complex (Trf4, Air1) | Yeast | [34,111] |
| Exosome complex (Rrp6, Dis3) | Yeast | [7,22,34] |
|  |  |  |
| **Splicing** |  |  |
| snRNP complexes (Prp31, Snu13, Snu114, Snu66) | Yeast | [22] |
| Yhc1 | Yeast | [22] |
| Mud2 | Yeast | [22] |
| SRSF1 | Chicken, mouse, human | [30,33] |
| RNPS1 | Chicken, human | [112] |
| OMCG1 | Mouse | [113] |
|  |  |  |
| **R-loop degradation** |  |  |
| RNase H1 | Bacteria, yeast, mouse, human | [22,31,34,36,114] |
| RNase H2 | Bacteria, yeast, mouse, human | [22,31,34,36,114] |
|  |  |  |
| **DNA topology** |  |  |
| Topoisomerase I | Bacteria, yeast, mouse, human | [11,33,50,115,116] |
| Topoisomerase IIIB | Mouse, human | [27] |
|  |  |  |
| **DNA and histone modifications** |  |  |
| H3S10p | Yeast, nematode, human | [26] |
| H3K9me2 | Yeast, human | [25,75] |
| H4R3me2a | Human, mouse | [27] |
| γH2A/γH2AX | Yeast, mouse, rat, human | [15,29,31,35,81,107,117] |
| CpG methylation (DNMT3B1) | Human | [18] |
| Psh1 | Yeast | [22] |
| Rpd3C(L) complex (Sin3, Sds3) | Yeast | [7,22,34] |
| AID | Yeast, mouse | [21,27,88,118] |
| BRE1A/B | Mouse | [81] |
|  |  |  |
| **DNA repair and genome maintenance** |  |  |
| Esc2 | Yeast | [22] |
| RecA/Rad51 | Bacteria, yeast | [7,119,120] |
| Rad52 | Yeast | [7,35] |
| Srs2 | Yeast | [7] |
| ATM | Mouse | [15] |
| TDP1 | Mouse | [15] |
| APTX | Mouse | [15] |
| BRCA2 | Human | [82] |
|  |  |  |
| **Single-strand DNA binding proteins** |  |  |
| AtNDX | Arabidopsis | [13] |
|  |  |  |
| **Other processes** |  |  |
| ORF57 | Kaposi’s sarcoma-associated herpes virus (KSHV) | [83] |
| ICP27 | Herpes simplex virus 1 (HSV1) | [83] |
| Sts1 | Yeast | [22] |
| Stb3 | Yeast | [34] |
|  |  |  |
| **RNA/DNA hybrid helicases** |  |  |
| NS3 | Hepatitis C & G virus | [121,122]* |
| UvsW | T4 Bacteriophage | [123] |
| Rho | Bacteria | [124,125]* |
| RecG | Bacteria | [120,126] |
| Cas3 | Bacteria | [127]* |
| DnaB/MCM complex | Bacteria, Archaea, yeast | [38]* |
| Pif1/PIF1 | Yeast, human | [37,128]* |
| Has1 | Yeast | [129]* |
| MLE/DHX9 | Drosophila, human | [130,131] * |
| SUV3 | Human | [132]* |
| WRN | Human | [133,134]* |
| DDX11 | Human | [135]* |
