# Supplementary material for: Out of Balance: R-loops in Human Disease
Source: PLoS Genet. 2014 Sep 18;10(9):e1004630. doi: 10.1371/journal.pgen.1004630 (PMC4169248; doi:10.1371/journal.pgen.1004630)
Supplement: Text S1 — Supplemental references. (DOCX) [file pgen.1004630.s002.docx]

**Supplemental references:**

106. Kaneko S, Chu C, Shatkin AJ, Manley JL (2007) Human capping enzyme promotes formation of transcriptional R loops in vitro. Proc Natl Acad Sci U S A 104: 17620-17625.

107. Morales JC, Richard P, Rommel A, Fattah FJ, Motea EA, et al. (2014) Kub5-Hera, the human Rtt103 homolog, plays dual functional roles in transcription termination and DNA repair. Nucleic Acids Res.

108. Gonzalez-Aguilera C, Tous C, Gomez-Gonzalez B, Huertas P, Luna R, et al. (2008) The THP1-SAC3-SUS1-CDC31 complex works in transcription elongation-mRNA export preventing RNA-mediated genome instability. Mol Biol Cell 19: 4310-4318.

109. Dominguez-Sanchez MS, Barroso S, Gomez-Gonzalez B, Luna R, Aguilera A (2011) Genome instability and transcription elongation impairment in human cells depleted of THO/TREX. PLoS Genet 7: e1002386.

110. Castellano-Pozo M, Garcia-Muse T, Aguilera A (2012) R-loops cause replication impairment and genome instability during meiosis. EMBO Rep 13: 923-929.

111. Gavalda S, Gallardo M, Luna R, Aguilera A (2013) R-loop mediated transcription-associated recombination in trf4Delta mutants reveals new links between RNA surveillance and genome integrity. PLoS One 8: e65541.

112. Li X, Niu T, Manley JL (2007) The RNA binding protein RNPS1 alleviates ASF/SF2 depletion-induced genomic instability. RNA 13: 2108-2115.

113. Houlard M, Artus J, Leguillier T, Vandormael-Pournin S, Cohen-Tannoudji M (2011) DNA-RNA hybrids contribute to the replication dependent genomic instability induced by Omcg1 deficiency. Cell Cycle 10: 108-117.

114. Tadokoro T, Kanaya S (2009) Ribonuclease H: molecular diversities, substrate binding domains, and catalytic mechanism of the prokaryotic enzymes. FEBS J 276: 1482-1493.

115. Masse E, Drolet M (1999) Escherichia coli DNA topoisomerase I inhibits R-loop formation by relaxing transcription-induced negative supercoiling. J Biol Chem 274: 16659-16664.

116. Marinello J, Chillemi G, Bueno S, Manzo SG, Capranico G (2013) Antisense transcripts enhanced by camptothecin at divergent CpG-island promoters associated with bursts of topoisomerase I-DNA cleavage complex and R-loop formation. Nucleic Acids Res 41: 10110-10123.

117. Paulsen RD, Soni DV, Wollman R, Hahn AT, Yee MC, et al. (2009) A genome-wide siRNA screen reveals diverse cellular processes and pathways that mediate genome stability. Mol Cell 35: 228-239.

118. Gomez-Gonzalez B, Aguilera A (2007) Activation-induced cytidine deaminase action is strongly stimulated by mutations of the THO complex. Proc Natl Acad Sci U S A 104: 8409-8414.

119. Kirkpatrick DP, Radding CM (1992) RecA protein promotes rapid RNA-DNA hybridization in heterogeneous RNA mixtures. Nucleic Acids Res 20: 4347-4353.

120. Hong X, Cadwell GW, Kogoma T (1995) Escherichia coli RecG and RecA proteins in R-loop formation. EMBO J 14: 2385-2392.

121. Gwack Y, Yoo H, Song I, Choe J, Han JH (1999) RNA-Stimulated ATPase and RNA helicase activities and RNA binding domain of hepatitis G virus nonstructural protein 3. J Virol 73: 2909-2915.

122. Du MX, Johnson RB, Sun XL, Staschke KA, Colacino J, et al. (2002) Comparative characterization of two DEAD-box RNA helicases in superfamily II: human translation-initiation factor 4A and hepatitis C virus non-structural protein 3 (NS3) helicase. Biochem J 363: 147-155.

123. Dudas KC, Kreuzer KN (2001) UvsW protein regulates bacteriophage T4 origin-dependent replication by unwinding R-loops. Mol Cell Biol 21: 2706-2715.

124. Brennan CA, Dombroski AJ, Platt T (1987) Transcription termination factor rho is an RNA-DNA helicase. Cell 48: 945-952.

125. Boudvillain M, Figueroa-Bossi N, Bossi L (2013) Terminator still moving forward: expanding roles for Rho factor. Curr Opin Microbiol 16: 118-124.

126. Vincent SD, Mahdi AA, Lloyd RG (1996) The RecG branch migration protein of Escherichia coli dissociates R-loops. J Mol Biol 264: 713-721.

127. Sinkunas T, Gasiunas G, Fremaux C, Barrangou R, Horvath P, et al. (2011) Cas3 is a single-stranded DNA nuclease and ATP-dependent helicase in the CRISPR/Cas immune system. EMBO J 30: 1335-1342.

128. Zhang DH, Zhou B, Huang Y, Xu LX, Zhou JQ (2006) The human Pif1 helicase, a potential Escherichia coli RecD homologue, inhibits telomerase activity. Nucleic Acids Res 34: 1393-1404.

129. Rocak S, Emery B, Tanner NK, Linder P (2005) Characterization of the ATPase and unwinding activities of the yeast DEAD-box protein Has1p and the analysis of the roles of the conserved motifs. Nucleic Acids Res 33: 999-1009.

130. Lee CG, Chang KA, Kuroda MI, Hurwitz J (1997) The NTPase/helicase activities of Drosophila maleless, an essential factor in dosage compensation. EMBO J 16: 2671-2681.

131. Chakraborty P, Grosse F (2011) Human DHX9 helicase preferentially unwinds RNA-containing displacement loops (R-loops) and G-quadruplexes. DNA Repair (Amst) 10: 654-665.

132. Shu Z, Vijayakumar S, Chen CF, Chen PL, Lee WH (2004) Purified human SUV3p exhibits multiple-substrate unwinding activity upon conformational change. Biochemistry 43: 4781-4790.

133. Suzuki N, Shimamoto A, Imamura O, Kuromitsu J, Kitao S, et al. (1997) DNA helicase activity in Werner's syndrome gene product synthesized in a baculovirus system. Nucleic Acids Res 25: 2973-2978.

134. Chakraborty P, Grosse F (2010) WRN helicase unwinds Okazaki fragment-like hybrids in a reaction stimulated by the human DHX9 helicase. Nucleic Acids Res 38: 4722-4730.

135. Hirota Y, Lahti JM (2000) Characterization of the enzymatic activity of hChlR1, a novel human DNA helicase. Nucleic Acids Res 28: 917-924.
